# Breast cancer in postmenopausal women is associated with an altered gut metagenome

**DOI:** 10.1186/s40168-018-0515-3

**Published:** 2018-08-06

**Authors:** Jia Zhu, Ming Liao, Ziting Yao, Wenying Liang, Qibin Li, Jianlun Liu, Huawei Yang, Yinan Ji, Wei Wei, Aihua Tan, Siyuan Liang, Yang Chen, Haisong Lin, Xiujuan Zhu, Shengzhu Huang, Jiarong Tian, Ruiqiang Tang, Qiuyan Wang, Zengnan Mo

**Affiliations:** 1 Center for Genomic and Personalized Medicine, Guangxi Medical University, Nanning, 530021 Guangxi China; 2 Department of Chemotherapy, The Affiliated Tumor Hospital of Guangxi Medical University, Nanning, 530021 Guangxi China; 3 Department of Breast Surgery, The Affiliated Tumor Hospital of Guangxi Medical University, Nanning, 530021 Guangxi China; 4 Clabee Genomics, Urban Garden Building, Bookstore Road, Luohu District, Shenzhen, 518000 Guangdong China; 5 Department of Colorectal Surgery, The First Affiliated Hospital of Guangxi Medical University, Nanning, 530021 Guangxi China; 6 Department of Breast Surgery, The First Affiliated Hospital of Nanchang University, Nanchang, 330000 Jiangxi China

**Keywords:** Breast cancer, Gut microbiota, Metagenomic analyses, Metabolism, Immunity

## Abstract

**Background:**

Increasing evidence suggests that gut microbiota play a role in the pathogenesis of breast cancer. The composition and functional capacity of gut microbiota associated with breast cancer have not been studied systematically.

**Methods:**

We performed a comprehensive shotgun metagenomic analysis of 18 premenopausal breast cancer patients, 25 premenopausal healthy controls, 44 postmenopausal breast cancer patients, and 46 postmenopausal healthy controls.

**Results:**

Microbial diversity was higher in breast cancer patients than in controls. Relative species abundance in gut microbiota did not differ significantly between premenopausal breast cancer patients and premenopausal controls. In contrast, relative abundance of 45 species differed significantly between postmenopausal patients and postmenopausal controls: 38 species were enriched in postmenopausal patients, including *Escherichia coli*, *Klebsiella sp_1_1_55*, *Prevotella amnii*, *Enterococcus gallinarum*, *Actinomyces* sp. *HPA0247*, *Shewanella putrefaciens*, and *Erwinia amylovora*, and 7 species were less abundant in postmenopausal patients, including *Eubacterium eligens* and *Lactobacillus vaginalis*. *Acinetobacter radioresistens* and *Enterococcus gallinarum* were positively but weakly associated with expression of high-sensitivity C-reactive protein; *Shewanella putrefaciens* and *Erwinia amylovora* were positively but weakly associated with estradiol levels. *Actinomyces* sp. *HPA0247* negatively but weakly correlated with CD3^+^CD8^+^ T cell numbers. Further characterization of metagenome functional capacity indicated that the gut metagenomes of postmenopausal breast cancer patients were enriched in genes encoding lipopolysaccharide biosynthesis, iron complex transport system, PTS system, secretion system, and beta-oxidation.

**Conclusion:**

The composition and functions of the gut microbial community differ between postmenopausal breast cancer patients and healthy controls. The gut microbiota may regulate or respond to host immunity and metabolic balance. Thus, while cause and effect cannot be determined, there is a reproducible change in the microbiota of treatment-naive patients relative to matched controls.

**Electronic supplementary material:**

The online version of this article (10.1186/s40168-018-0515-3) contains supplementary material, which is available to authorized users.

## Background

The human gut harbors thousands of bacterial species, together making up a population as large as 10^13–14^ microbes, which encode 150-fold more genes than the human genome [1–3]. The gut microbiota is composed of a large number of anaerobic microorganisms, predominantly Bacteroidetes and Firmicutes [4, 5], which are affected by a multitude of factors including host genetics, lifestyle, and environment. The microbiota plays important roles in maintaining an intestinal mucosal barrier, antagonizing the colonization of pathogenic microorganisms, and contributing to metabolism and immune homeostasis [6].

The gut microbiota has been linked to various diseases, such as inflammatory bowel disease [7, 8], obesity [9], diabetes [10, 11], rheumatoid arthritis [12], atopic manifestations [13], liver cirrhosis [14], cardiovascular diseases [15], mental diseases [16, 17], and colorectal cancer [18, 19]. The gut microbiota exert an influence on both local and systemic metabolism and immunity [20, 21], and alterations of gut microbiota have been associated with extra-intestinal cancers including hepatocellular carcinoma, to which they may contribute by triggering chronic inflammation and altering microenvironment and metabolism [22, 23].

Increasing evidence suggests that microbe-host interactions have the potential to influence or serve as a biomarker of breast cancer pathogenesis [24, 25]. A comparison of 11 breast cancer patients and 7 healthy controls revealed differences in the gut microbiota, with *Clostridia*, *Enterobacterium*, *Lactobacilli*, *Bacteroides*, and *Escherichia coli* enriched in patients [24]. A comparison of 48 postmenopausal breast cancer case patients and 48 healthy controls [25] revealed an altered, less diverse gut microbiota in patients: *Clostridiales*, *Clostridiaceae*, *Faecalibacterium*, and *Ruminococcaceae* were enriched in patients, while *Dorea* and *Lachnospiraceae* were relatively less abundant in patients. Among controls, microbiota diversity correlated with total estrogen levels, suggesting that gut microbiota may be implicated in breast cancer by responding to or affecting estrogen metabolism.

Those previous studies have provided useful insights into the potential response of gut microbiota in breast cancer, but they have not been able to comprehensively catalog the taxonomies of the microbiota because they have relied on only biochemical analysis or 16S rRNA gene sequencing. In addition, previous studies did not explore the functional capacity of the gut microbiota in patients with breast cancer, which could provide more mechanistic insights into the role of the gut microbiota in this disease. As a result, how the gut microbiota and their biochemical and metabolic products change in breast cancer is unclear.

To address these questions, we used shotgun metagenomic analysis to compare the gut microbial community and its functional capabilities between breast cancer patients and healthy controls.

## Methods

### Subjects

The study was approved by the Ethics Committee of the Affiliated Tumor Hospital of Guangxi Medical University (Nanning, China). Fecal samples were collected from 18 premenopausal breast cancer patients, 25 premenopausal healthy controls, 44 postmenopausal breast cancer patients, and 46 postmenopausal healthy controls (Table 1). All patients with breast cancer were diagnosed by pathological examination at the Affiliated Tumor Hospital, and healthy controls were recruited from the Medical Examination Center of the First Affiliated Hospital of Guangxi Medical University. Controls were free of breast cancer at medical examination. None of the study subjects had diarrhea, diabetes, ulcerative colitis, Crohn’s disease, or other infectious diseases. No subjects took antibiotics, steroid hormones, Chinese herbal medicine (including oral, intramuscular, or intravenous injection), or probiotics such as yogurt during the 3 months before fecal sample collection. Breast cancer patients did not receive chemotherapy, radiation, or surgery prior to fecal sample collection.

**Table 1:** Demographic characteristics of participants

	Premenopausal group		*P* value	Postmenopausal group		*P* value
Characteristic	Cases *N* = 18	Controls *N* = 25		Cases *N* = 44	Controls *N* = 46	
Age (m ± sd)	37.06 ± 5.23	35.52 ± 6.02	0.389	57.45 ± 7.41	56.89 ± 6.41	0.7
BMI (m ± sd)	22.95 ± 3.88	23.01 ± 1.95	0.952	23.64 ± 2.77	23.97 ± 2.50	0.559
Age at menopause, years	–	–	–	49.39 ± 3.15	48.70 ± 2.87	0.29
Ethnicity, *n* (%)			0.607			0.985
Han	12 (66.7)	20 (80.0)		33 (75.0)	34 (73.9)	
Zhuang	5 (27.8)	4 (16.0)		8 (18.2)	9 (19.6)	
Other	1 (5.5)	1 (4.0)		3 (6.8)	3 (6.5)	

Fecal samples were freshly collected from individuals and transported to the laboratory on ice. Samples were stored at − 80 °C until extraction. Bacterial DNA was extracted from fecal samples using the QIAampDNA Stool MiniKit (Qiagen, Hilden, Germany) according to the manufacturer’s instructions.

### Metagenomic DNA sequencing and annotation

All samples were sequenced on the Illumina HiSeq × 10 platform. A paired-end library was constructed with 350-bp inserts for each sample. Low-quality reads and reads mapping to human DNA were removed from the raw data. For taxonomic assignments, the high-quality reads from each sample were aligned against the integrated reference catalog of the human gut microbiome (IGC) by bowtie2 using the criterion of “identity > 90%,” genes from the existing reference gene catalog IGC inherited their original taxonomic annotation, and the relative abundance of a taxon was calculated from the relative abundance of its genes [26, 27]. During KO profiling, genes from IGC inherited their original KO annotation, and KO abundance was calculated by summing the relative abundance of genes annotated to the same KO [26, 27].

### Quantification of virulence factors and pathogen-host interaction genes

Genes in the catalog were aligned against proteins in the Virulence Factors of Pathogenic Bacteria database [28] using BLAST (version 2.2.24) set to default parameters, except that *-p blastx -e 1e-5 -F F -a 4 -m 8*. We selected the matches with the highest-scoring annotated hit containing identity > 40% and high-scoring segment pair scoring > 60 bits. The relative abundance of a virulence factor was calculated by summing the relative abundance of genes annotated to a feature. Genes in the gene catalog were aligned against the proteins in the Pathogen-Host Interactions database [29] using BLAST (version 2.2.24) set to default parameters except that *-p blastx -e 1e-5 -F F -a 4 -m 8*. We selected the matches with the highest-scoring annotated hits containing an identity > 40% and high-scoring segment pair scoring > 60 bits. The relative abundance of an interaction gene was calculated by summing the abundance of genes annotated to a feature.

### Gut microbiota diversity

Based on the species profile, we calculated the within-sample (alpha) diversity to estimate gut microbiota richness and evenness based on the Shannon index and Chao1 index [30]. High alpha diversity indicates high diversity of gut microbiota within a sample. Between-sample differences in microbial composition (beta diversity) were assessed in terms of the Jensen-Shannon divergence (JSD) [31]. The JSD was calculated by the following steps: (1) We first calculated JSD between each two samples within one group. (2) The mean of all JSD values between a sample and others within one group was computed (the mean value represented the similarity of the sample to others). We compared the mean JSD values to find if the beta diversity is different or not among the groups.

### Enterotyping

Samples were clustered based on relative genus abundances using JSD distance and the “partitioning around medoids” (PAM) clustering algorithm. The Calinski-Harabasz (CH) index was used to calculate the optimal number of clusters [32]. Principal component analysis was used to visualize the taxonomic drivers of clusters.

### Statistical analysis

Demographics were compared among groups using Student’s *t* test or the chi-square test in SPSS 16.0 software (IBM, Chicago, IL, USA). R software (version 3.3.2) was used to perform other analyses. The Wilcoxon rank sum test was used to identify significant differences in abundance of genes, genera, virulence factors, interaction genes, and KOs. Differentially enriched pathways and modules were identified according to their reporter score from the *Z* scores of significant KOs. A module with a reporter score of *Z* > 1.6 was defined as differentially enriched [33, 34]. *P* values were adjusted based on the false discovery rate (FDR) using the method of Benjamini and Hochberg [35]. Permutational multivariate analysis of variance (PERMANOVA) using the “adonis” function in the R Vegan package was performed to assess effects of phenotype on gene/taxa profiles. The R package “ade4,” which involves instrumental principal component analysis [36], was used to visualize the taxonomic drivers of clusters during enterotyping. The “pheatmap” package (version 1.0.8) was used to generate heat maps, and the clustering method used in “pheatmap” function was “correlation.” Spearman’s rank correlation was used to find correlations of metagenomic features and clinical indices.

A species-based classifier was trained using the random forest package in R. A tenfold cross-validation was performed on a random forest model using the relative species abundance profile. The minimum error was calculated using fivefold cross-validation with the “rfcv” function, and the minimum error plus the s.d. at that point was used as the cutoff. The optimal number of species was selected by cross-validation with one SE rule. The case probability was calculated using this set of species and a receiver operating characteristic (ROC) curve within the pROC package in R. The model was tested on the testing set, and the prediction error was determined [37]. Differentially enriched genes were identified using the Wilcoxon rank test, and adjusted *P* values were estimated using the R package “*q* value” (version 2.2.2). All differentially enriched genes (*q* value < 0.05) were annotated to the butanoate metabolism pathways (using their original KO annotation which was inherited from the integrated reference catalog of the human gut microbiome database).

## Results

### Taxonomic characterization of gut microbiota in breast cancer patients and healthy controls

A total of 133 stool samples were sequenced from premenopausal breast cancer patients (*n* = 18), premenopausal healthy controls (*n* = 25), postmenopausal breast cancer patients (*n* = 44), and postmenopausal healthy controls (*n* = 46). The premenopausal breast cancer patients and controls were similar for age, BMI, and ethnicity (*P* > 0.05, Table 1); the postmenopausal breast cancer patients and controls were similar for age, BMI, age at menopause, and ethnicity (*P* > 0.05, Table 1). A total of 965 million 150-bp paired-end reads were generated, with an average (s.d.) of 7.25 ± 1.13 million reads for each sample. After quality control, we obtained 902 million high-quality reads free of adaptor and human DNA contaminants, with an average (s.d.) of 6.78 ± 1.08 million reads per sample (Additional file 1: Table S1).

To determine whether the sequencing adequately captured the gene diversity of the gut microbiota, rarefaction analysis was performed. The curves in all samples were near saturation, suggesting that the sequencing depth was sufficient to capture most gene diversity (Additional file 2: Figure S1).

**Fig. 1:**
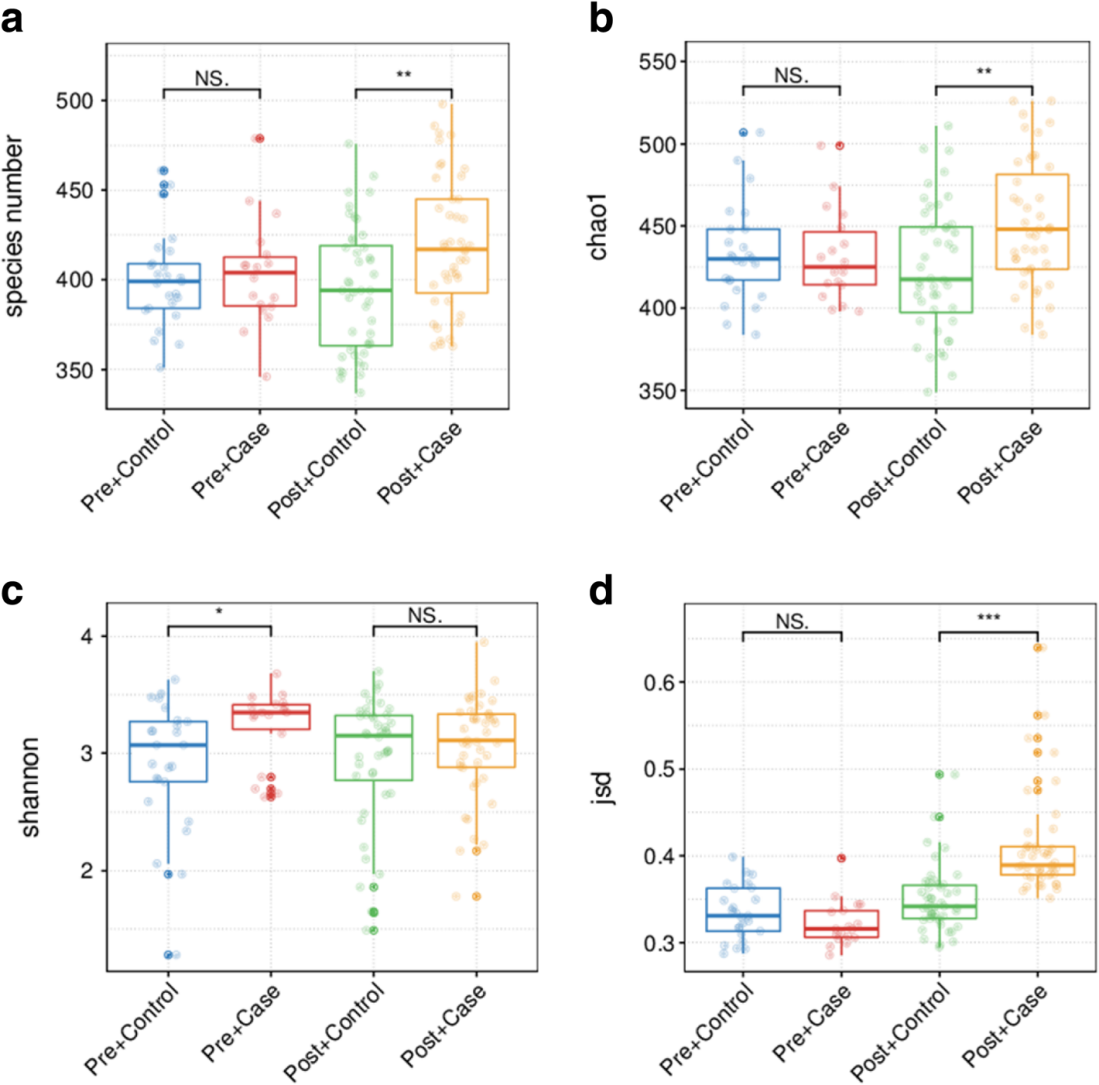
Diversity of gut microbiota in breast cancer patients and healthy controls. **a** Total number of species in the four groups. **b**, **c** Alpha diversity of the four cohorts at species level, measured in terms of the Chao1 index and Shannon index. **d** Beta diversity of the four cohorts at species level. Each dot refers to a sample; if a sample has a high average JSD value, it indicates that the gut microbiota community structure of this sample is very different. Furthermore, if most samples of a group have high average JSD values, it indicates that the between-sample variability of the group is high. NS non-significant. *P < 0.05, **P < 0.01, ***P < 0.001

A similar number of species was detected in premenopausal breast cancer patients and premenopausal healthy controls (*P* = 0.767, Wilcoxon rank sum test, Fig. 1a). Based on the species profile, we calculated the within-sample (alpha) diversity to estimate gut microbiota richness and evenness based on the Shannon index and Chao1 index. The mean Chao1 index was similar between premenopausal breast cancer patients and premenopausal controls (*P* = 0.777, Wilcoxon rank sum test, Fig. 1b). The mean Shannon index was higher for premenopausal patients than premenopausal controls (*P* = 0.027, Wilcoxon rank sum test, Fig. 1c).

In contrast, the between-sample variability (beta diversity) of the gut microbiota community structure tended to be lower in premenopausal patients than in premenopausal controls (*P* = 0.056, Fig. 1d).

The number of species was significantly higher in postmenopausal breast cancer patients than in postmenopausal controls (*P* = 0.003, Wilcoxon rank sum test, Fig. 1a). Consistently, the mean Chao1 index was higher in postmenopausal patients than in postmenopausal controls (*P* = 0.007, Wilcoxon rank sum test, Fig. 1b). However, mean Shannon index was similar between postmenopausal breast cancer patients and postmenopausal controls (*P* = 0.502, Wilcoxon rank sum test, Fig. 1c). Beta diversity was higher for postmenopausal patients than postmenopausal controls (*P* < 0.001, Fig. 1d).

Previous studies have suggested that the human gut microbiome can be assigned to several robust enterotypes [38, 39]. To group the breast cancer patients and control samples into enterotype clusters, we applied the PAM method using JSD for the relative abundance of genera.

The optimal number of enterotypes was 2 as indicated by the CH index (Additional file 2: Figure S2a). Principal component analysis was used to cluster the samples of the four groups into two enterotypes (Additional file 2: Figure S2b). Enterotype 1 had a relatively high level of *Bacteroides*, enterotype 2, a relatively high level of *Prevotella* (Additional file 2: Figure S2c). These two enterotypes have been observed in European and Chinese populations [38, 39]. However, we found no significant relationship between enterotype and breast cancer disease status, either when we compared premenopausal patients with premenopausal controls (*P* = 0.141) or when we compared postmenopausal patients with postmenopausal controls (*P* = 0.445; Fisher’s exact test in both cases; Additional file 1: Table S2; Additional file 2: Figure S2d).

**Fig. 2:**
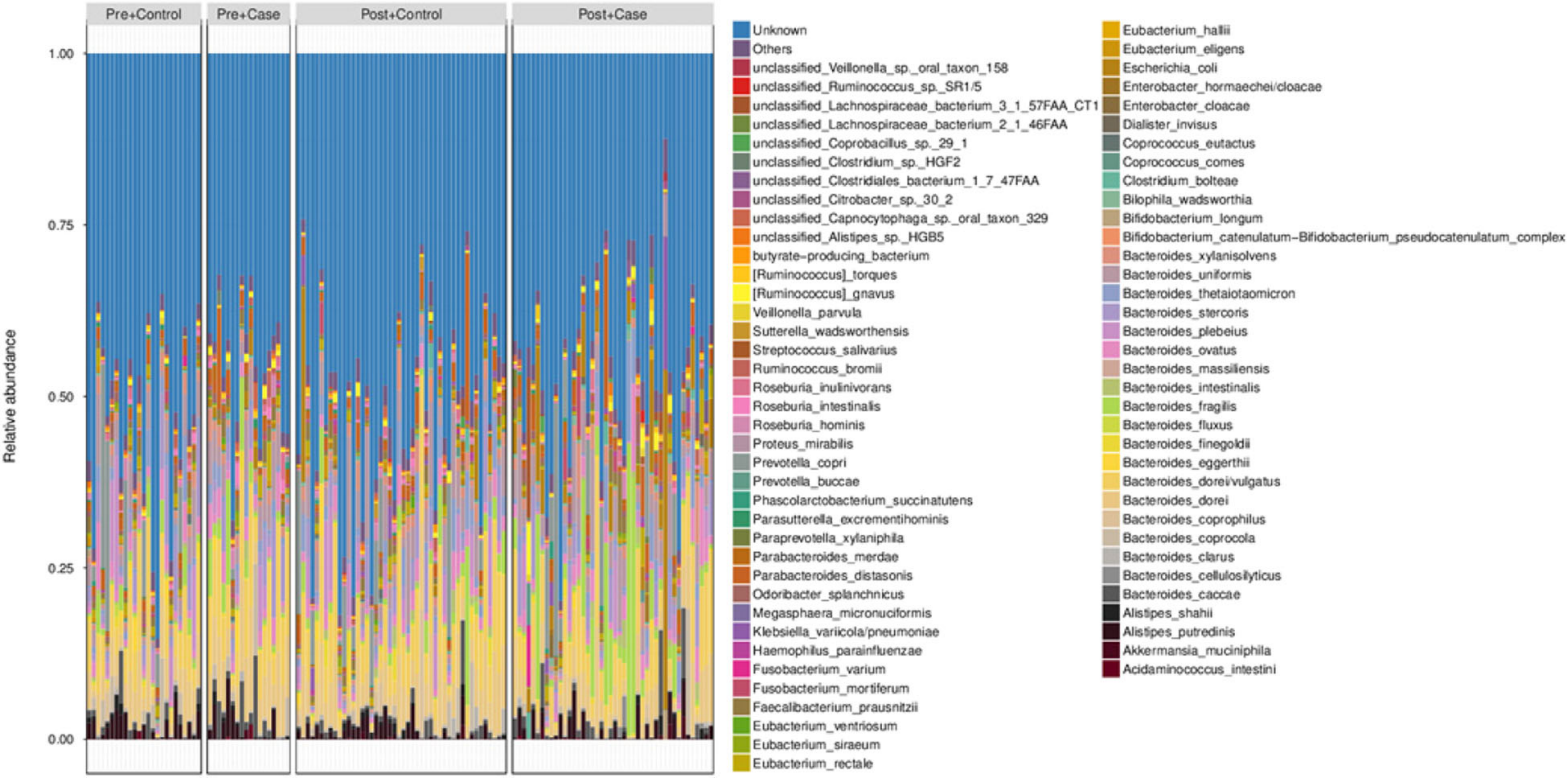
Relative abundance of the gut microbiota in the four groups at species level

To further explore features of the gut microbial community in breast cancer patients, we compared the relative abundances of species between patients and controls. The taxonomic assignment for the metagenomic data was carried out using bowtie. The relative abundance of gut microbiota was calculated by summing the abundance of genes. Relative abundance of the gut microbiota in the four groups at the species level is shown in Fig. 2.

**Fig. 3:**
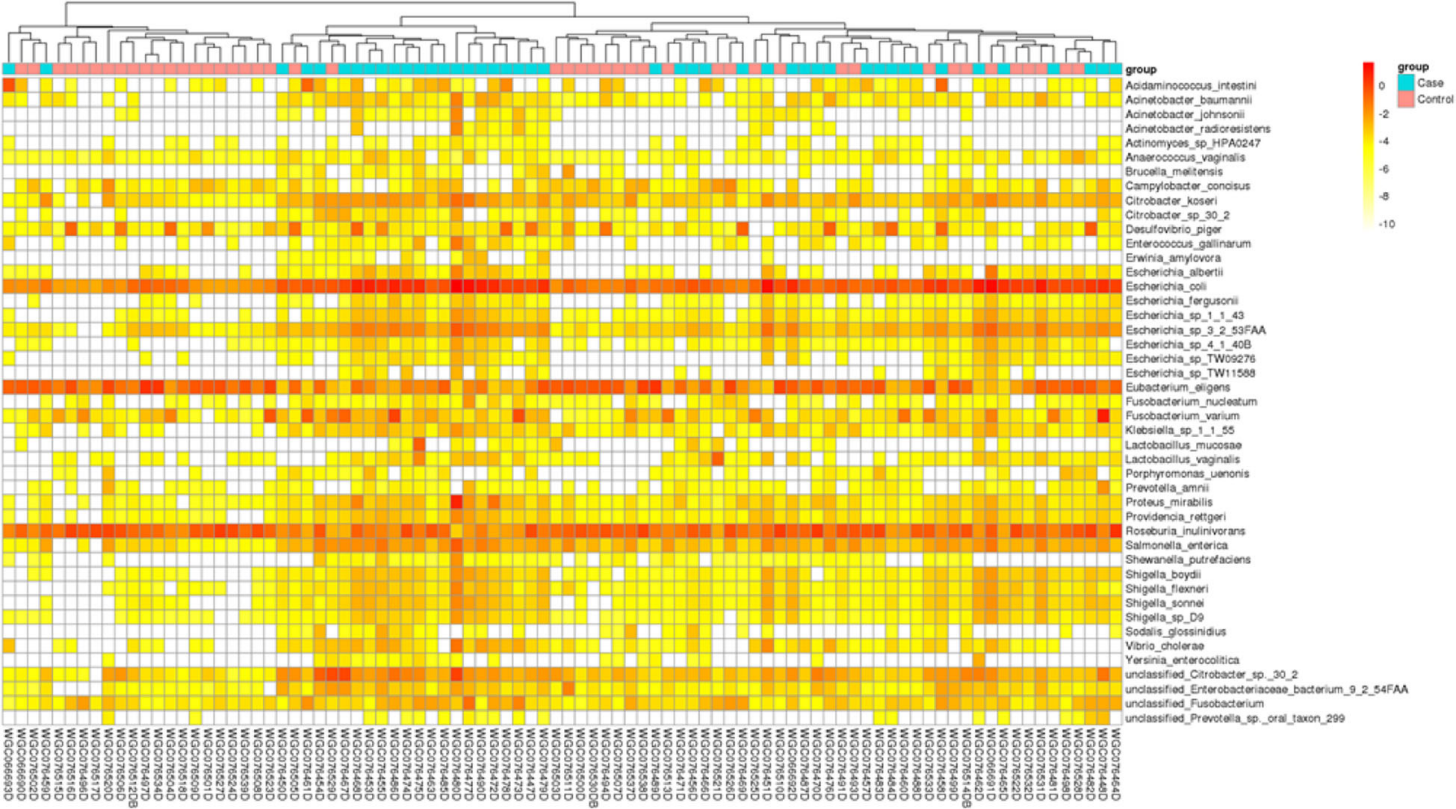
Relative abundance of 45 species differing significantly between postmenopausal breast cancer patients and postmenopausal healthy controls

There was no significant difference in gut microbiota species between premenopausal breast cancer patients and premenopausal healthy controls (*q* value > 0.05, Wilcoxon rank sum test; Additional file 1: Table S3). In contrast, 45 species differed significantly between postmenopausal patients and postmenopausal controls: 38 species were enriched in patients, including *Escherichia coli*, *Klebsiella sp_1_1_55*, and *Prevotella amnii*, while 7 species were reduced in patients, including *Porphyromonas uenonis*, *Eubacterium eligens*, and *Lactobacillus vaginalis* (*q* value < 0.05; Table 2; Additional file 1: Table S4, Fig. 3). PERMANOVA analysis showed that breast cancer status, menopause status, and age were significant factors for explaining the variation in the examined gut microbial samples (*P* < 0.05; Additional file 1: Table S5).

**Table 2:** Relative abundance of the different species between postmenopausal breast cancer patients and postmenopausal healthy controls

	*P* value	*q* value	Control_mean	Control_sd	Case_mean	Case_sd
Increased in postmenopausal breast cancer patients						
*Escherichia_coli*	1.45E−07	4.77E−05	1.04E−02	4.34E−02	3.74E−02	8.18E−02
*Shigella_sp_D9*	1.86E−07	4.77E−05	1.80E−06	8.35E−06	5.36E−06	1.50E−05
*Escherichia_sp_3_2_53FAA*	3.03E−07	4.77E−05	6.92E−05	2.96E−04	2.40E−04	5.14E−04
*Shigella_sonnei*	3.01E−07	4.77E−05	4.38E−06	1.94E−05	1.46E−05	3.42E−05
*Escherichia_sp_1_1_43*	6.49E−07	8.17E−05	2.53E−06	1.11E−05	8.11E−06	1.84E−05
*Proteus_mirabilis*	1.53E−06	1.61E−04	1.94E−06	5.26E−06	1.46E−03	9.47E−03
*Shigella_boydii*	2.17E−06	1.95E−04	3.07E−06	1.29E−05	1.01E−05	2.27E−05
*Vibrio_cholerae*	2.66E−06	2.09E−04	1.18E−06	3.46E−06	2.95E−05	1.17E−04
*Escherichia_fergusonii*	1.25E−05	8.72E−04	3.52E−07	1.68E−06	1.67E−06	3.34E−06
*Escherichia_sp_4_1_40B*	1.86E−05	1.06E−03	1.60E−06	7.36E−06	4.13E−06	1.06E−05
*Shigella_flexneri*	1.75E−05	1.06E−03	2.86E−06	1.20E−05	1.20E−05	4.58E−05
*Acinetobacter_baumannii*	5.39E−05	2.83E−03	1.73E−06	5.39E−06	1.25E−05	5.13E−05
*Escherichia_sp_TW09276*	8.01E−05	3.88E−03	6.60E−07	3.13E−06	1.95E−06	4.75E−06
*Actinomyces_sp_HPA0247*	9.98E−05	4.42E−03	2.65E−08	9.16E−08	1.67E−07	3.52E−07
*Acinetobacter_johnsonii*	1.23E−04	4.46E−03	4.28E−08	1.64E−07	5.64E−06	3.32E−05
*Providencia_rettgeri*	1.28E−04	4.46E−03	2.39E−06	7.06E−06	1.05E−05	3.25E−05
*Lactobacillus_mucosae*	1.27E−04	4.46E−03	4.19E−07	2.14E−06	5.36E−05	3.52E−04
*unclassified_Citrobacter_sp._30_2*	2.12E−04	7.02E−03	1.93E−04	1.12E−03	6.39E−04	2.27E−03
*Citrobacter_sp_30_2*	2.58E−04	8.11E−03	7.53E−07	3.99E−06	2.59E−06	7.86E−06
*Porphyromonas_uenonis*	4.41E−04	1.32E−02	7.70E−07	2.88E−06	1.19E−06	3.73E−06
*Citrobacter_koseri*	4.81E−04	1.38E−02	2.49E−05	5.29E−05	8.09E−05	1.87E−04
*Desulfovibrio_piger*	5.27E−04	1.44E−02	1.14E−04	3.37E−04	3.15E−04	7.13E−04
*Klebsiella_sp_1_1_55*	7.03E−04	1.62E−02	3.14E−06	8.91E−06	7.58E−06	1.77E−05
*Enterococcus_gallinarum*	6.76E−04	1.62E−02	2.62E−07	8.17E−07	1.48E−05	8.63E−05
*Salmonella_enterica*	6.49E−04	1.62E−02	4.29E−05	9.78E−05	1.49E−04	4.36E−04
*Erwinia_amylovora*	8.12E−04	1.71E−02	1.23E−08	5.96E−08	8.77E−07	3.02E−06
*Sodalis_glossinidius*	8.15E−04	1.71E−02	3.44E−07	2.09E−06	1.55E−06	5.56E−06
*Acinetobacter_radioresistens*	8.57E−04	1.74E−02	1.15E−08	7.82E−08	5.84E−06	3.50E−05
*Fusobacterium_varium*	1.00E−03	1.97E−02	1.62E−04	6.01E−04	2.49E−03	1.31E−02
*Acidaminococcus_intestini*	1.06E−03	2.02E−02	1.14E−06	3.45E−06	1.99E−04	8.37E−04
*Prevotella_amnii*	1.28E−03	2.37E−02	6.72E−07	3.56E−06	3.56E−06	1.87E−05
*Yersinia_enterocolitica*	1.32E−03	2.38E−02	4.48E−08	2.69E−07	7.96E−07	4.09E−06
*unclassified_Fusobacterium*	1.89E−03	2.90E−02	7.79E−06	1.68E−05	4.58E−05	1.52E−04
*unclassified_Prevotella_sp._oral_taxon_299*	1.79E−03	2.90E−02	2.17E−08	1.18E−07	4.34E−07	1.92E−06
*Anaerococcus_vaginalis*	1.81E−03	2.90E−02	1.11E−06	4.26E−06	1.46E−06	2.81E−06
*Shewanella_putrefaciens*	1.84E−03	2.90E−02	7.68E−08	2.12E−07	3.60E−06	2.05E−05
*Fusobacterium_nucleatum*	2.53E−03	3.79E−02	3.40E−07	8.82E−07	3.33E−06	1.74E−05
*Escherichia_sp_TW11588*	3.22E−03	4.61E−02	2.76E−07	1.75E−06	1.25E−06	3.63E−06
Decreased in postmenopausal breast cancer patients						
*Eubacterium_eligens*	1.05E−04	4.42E−03	4.02E−03	5.95E−03	1.46E−03	3.84E−03
*Escherichia_albertii*	7.21E−04	1.62E−02	1.28E−05	8.26E−05	9.96E−06	4.44E−05
*Campylobacter_concisus*	6.80E−04	1.62E−02	1.51E−05	4.24E−05	2.05E−06	4.30E−06
*unclassified_Enterobacteriaceae_bacterium_9_2_54FAA*	1.45E−03	2.53E−02	1.04E−05	4.38E−05	1.00E−05	1.78E−05
*Roseburia_inulinivorans*	1.89E−03	2.90E−02	3.69E−03	3.72E−03	2.64E−03	5.39E−03
*Brucella_melitensis*	3.15E−03	4.61E−02	1.83E−06	1.23E−05	5.73E−07	1.49E−06
*Lactobacillus_vaginalis*	3.44E−03	4.81E−02	4.32E−05	2.89E−04	7.07E−06	2.97E−05

### Identification of postmenopausal breast cancer patients based on gut microbiota

**Fig. 4:**
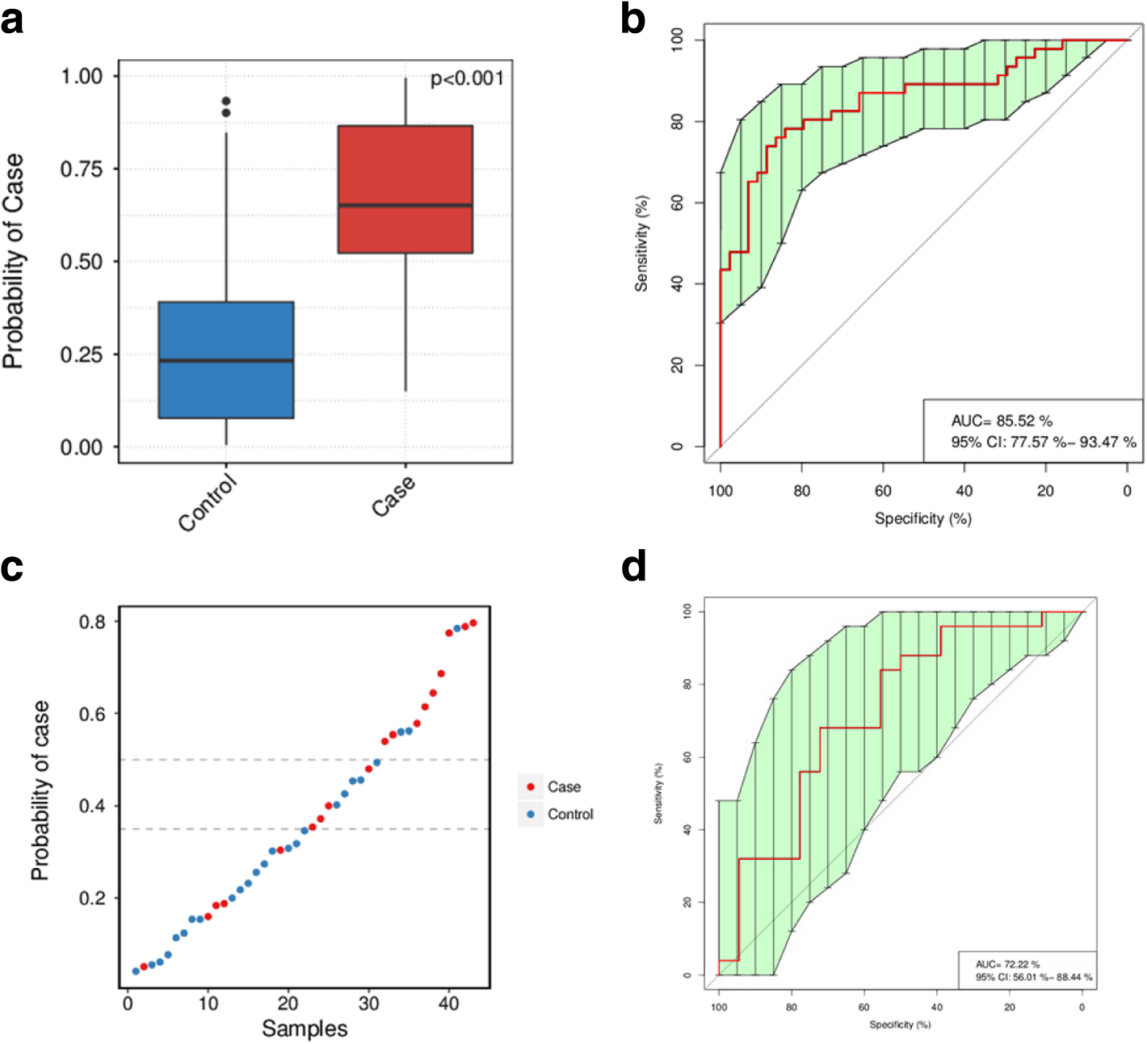
Classification to differentiate samples from postmenopausal breast cancer patients or postmenopausal healthy controls. **a** Probability of postmenopausal breast cancer in the training set. **b** ROC curves for the training set. The AUC was 87.25% (95% CI 79.82–94.68%). Classification of the test set consisted of 18 premenopausal breast cancer patients (red) and 25 premenopausal healthy controls (blue). **c** Classification of each sample. We used two cutoffs to assign the samples into three groups: 50% cases were classified into “Case” (probability of case > 50%), and 68% controls were classified into “Not case” (probability of case < 35%). Four cases and five controls stayed in “Uncertain.” **d** ROC for the test set. The AUC is 72% and the 95% CI is 56.01–88.44%

To illustrate the potential diagnostic value of gut microbiota for breast cancer in postmenopausal women, we used a random forest classifier in an attempt to detect breast cancer samples from among a mixture of samples from postmenopausal patients and healthy controls. Tenfold cross-validation was repeated for five times with a training set consisting of 44 postmenopausal patients and 46 postmenopausal controls; 14 optimal species markers were selected, including *Escherichia coli*, *Shigella sp_D9*, *Eubacterium eligens*, *Proteus mirabilis*, and *Fusobacterium varium* (Additional file 2: Figure S6, Additional file 1: Table S6). ROC curves for the training set showed a remarkable performance in the training set when discriminating between postmenopausal breast cancer patients and postmenopausal healthy controls by specificity and sensitivity; the area under receiver operating curve (AUC) was 85.52; and 95% confidence interval (CI) is 77.57–93.47% (Fig. 4b). Next, we tested the same markers for their ability to detect breast cancer among 43 samples not used during training, comprising 18 premenopausal breast cancer patients and 25 premenopausal healthy controls. The AUC was 72% (95% CI 56.01–88.44%; Fig. 4d).

### Quantification of virulence factors and pathogen-host interaction genes in the gut microbiota of postmenopausal breast cancer patients and postmenopausal healthy controls

To analyze proteins encoded by genes in gut microbiota, genes in the catalog were aligned against proteins in the Pathogen-Host Interactions (PHI) database and in the Virulence Factors of Pathogenic Bacteria database. The relative abundances of genes encoding virulence factors or pathogen-host interaction were calculated by summing the abundances of genes annotated to a feature.

**Fig. 5:**
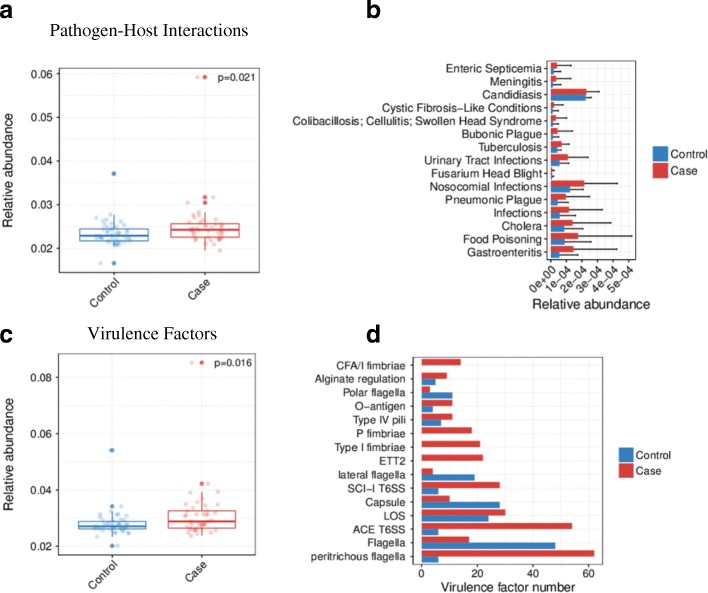
Relative abundance of genes encoding virulence factors and pathogen-host interactions in postmenopausal breast cancer patients and postmenopausal controls. **a** Relative abundance of pathogen-host interaction genes in the two groups. **b** The top 15 representation of Pathogen-Host Interactions genes in the two groups and their implication in human diseases. **c** Relative abundance of virulence factor genes in the two groups. **d** Relative abundance of the top 15 virulence factor genes in the two groups

PHI genes were relatively more abundant in postmenopausal breast cancer patients than in postmenopausal controls (*P* = 0.021, Fig. 5a). The top 15 representation of Pathogen-Host Interactions genes implicated in several human diseases, such as urinary tract infections, other infections, and tuberculosis (*q* value < 0.05, Wilcoxon rank sum test, Fig. 5b; Additional file 1: Table S7).

Virulence factors were relatively more abundant in postmenopausal patients than in controls (*P* = 0.016, Fig. 5c); these factors included LOS glycosyltransferase, peritrichous flagella, and type I fimbriae (*q* value < 0.05, Wilcoxon rank sum test, Fig. 5d; Additional file 1: Table S8).

### Association between gut microbiota and clinical indices

**Fig. 6:**
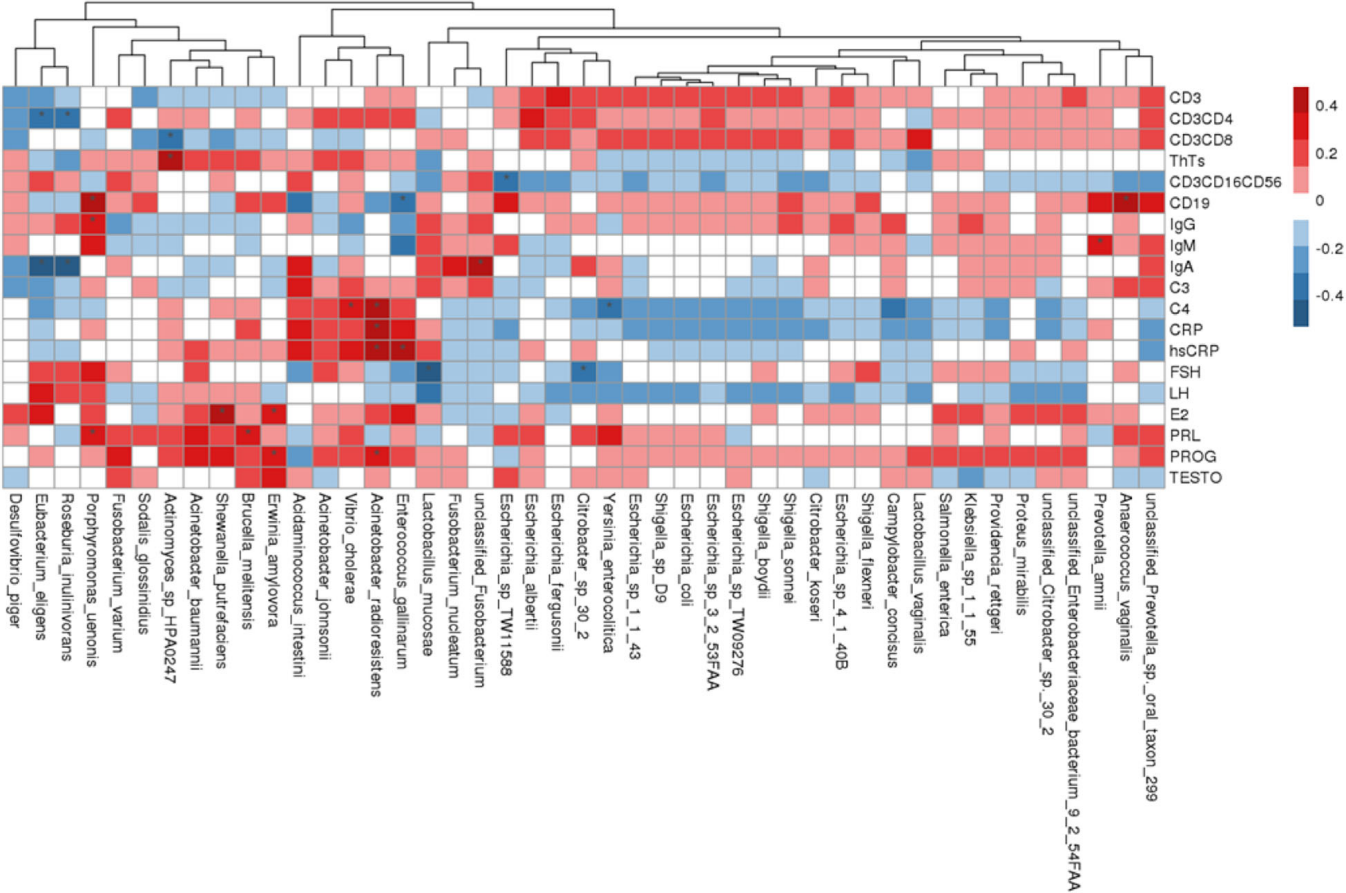
Correlation between gut microbiota species and clinical indices of breast cancer. Spearman’s rank correlation coefficient is indicated using a color gradient: red indicates positive correlation; blue, negative correlation. *P < 0.05

Next, we asked whether the 45 microbial species differing between postmenopausal breast cancer patients and postmenopausal controls correlated with well-established clinical indices of breast cancer based on Spearman correlation analysis (Fig. 6). Two gut microbiota species, *Acinetobacter radioresistens* (Spearman rho = 0.413, *P* = 0.015, *q* value > 0.05) and *Vibrio cholerae* (Spearman rho = 0.349, *P* = 0.043, *q* value > 0.05), positively but weakly correlated with C4 levels, whereas *Yersinia enterocolitica* (Spearman rho = − 0.345, *P* = 0.046, *q* value > 0.05) negatively but weakly correlated with C4 levels. Two species, *Acinetobacter radioresistens* (Spearman rho = 0.442, *P* = 0.009, *q* value > 0.05) and *Enterococcus gallinarum* (Spearman rho = 0.386, *P* = 0.024, *q* value > 0.05), positively but weakly correlated with levels of high-sensitivity C-reactive protein (hsCRP). *Anaerococcus vaginalis* (Spearman rho = 0.48, *P* = 0.002, *q* value > 0.05) and *Porphyromonas uenonis* (Spearman rho = 0.42, *P* = 0.009, *q* value > 0.05) positively but weakly correlated with CD19 levels, while *Enterococcus gallinarum* (Spearman rho = − 0.351, *P* = 0.031, *q* value > 0.05) negatively but weakly correlated with CD19 levels. *Shewanella putrefaciens* (Spearman rho = 0.379, *P* = 0.025, *q* value > 0.05) and *Erwinia amylovora* (Spearman rho = 0.351, *P* = 0.039, *q* value > 0.05) positively but weakly correlated with estradiol levels. *Actinomyces* sp. *HPA0247* (Spearman rho = − 0.384, *P* = 0.017, *q* value > 0.05) negatively but weakly correlated with CD3^+^CD8^+^ T cell numbers. All these gut flora species were enriched in postmenopausal breast cancer patients relative to controls. Conversely, *Eubacterium eligens* was enriched in controls relative to patients, yet it negatively but weakly correlated with CD3^+^CD4^+^ T cell numbers (Spearman rho = − 0.349, *P* = 0.032, *q* value > 0.05) and IgA levels (Spearman rho = − 0.532, *P* = 0.001, *q* value > 0.05).

### Metabolic functions of the gut microbiota in breast cancer patients and healthy controls

We explored functional features of the gut microbiota across the four groups in our study by annotating the gene catalog based on the KEGG modules. Modules differing between breast cancer patients and healthy controls with a reporter score > 1.6 were identified.

**Fig. 7:**
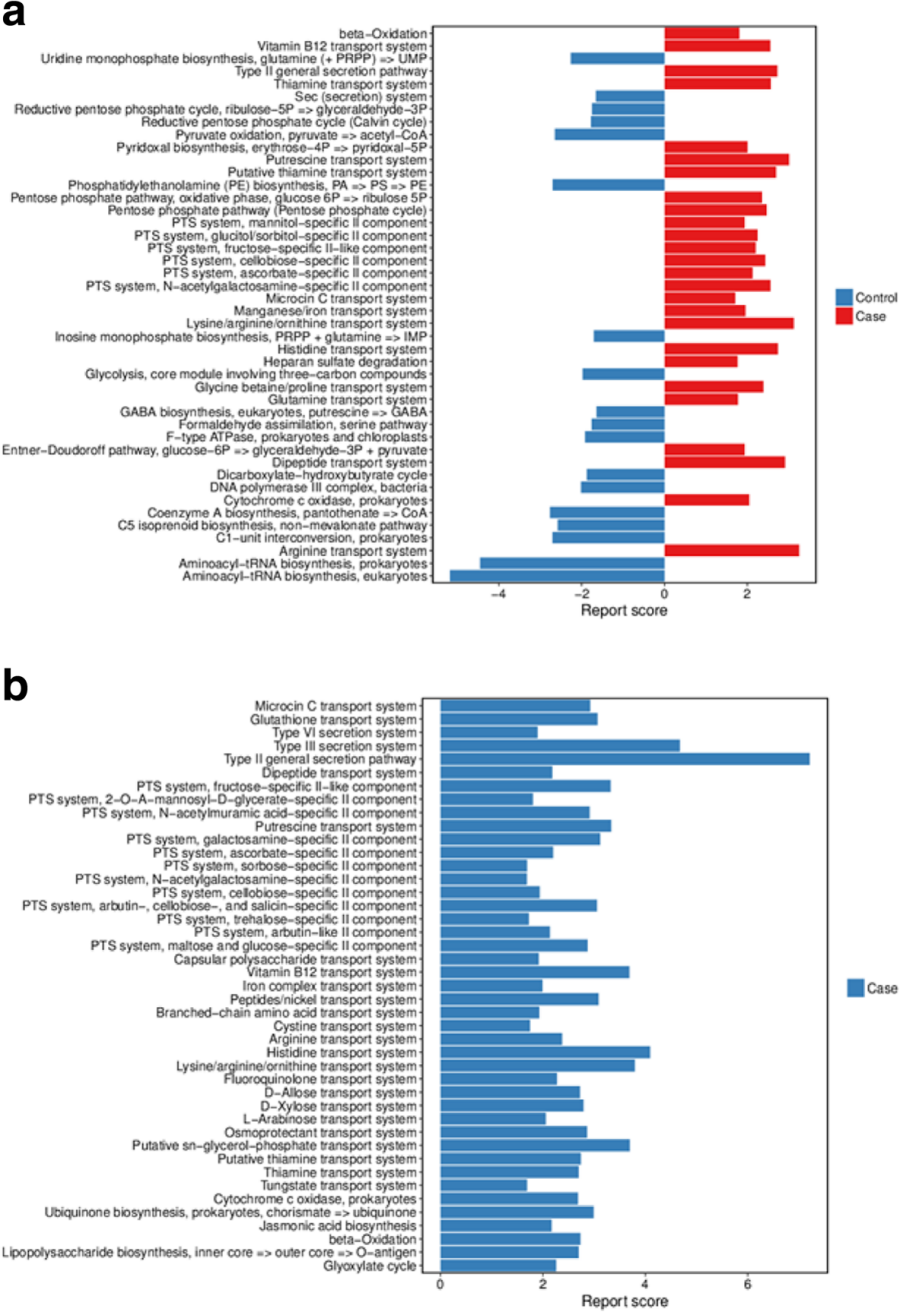
Functions of genes expressed in gut microbiota in pre- and postmenopausal breast cancer patients and healthy controls. **a** Gene functions in gut microbiota in premenopausal patients and controls; 26 KEGG modules were enriched in patients (red), and 18 were enriched in controls (blue). **b** Gene functions in gut microbiota in postmenopausal patients and controls; 43 KEGG modules were enriched in patients (blue)

Among premenopausal women, 44 KEGG modules were significantly different between patients and controls (*q* value < 0.05, Wilcoxon rank sum test, Fig. 7a; Additional file 1: Table S9). Modules enriched in patients included the PTS system, secretion system, vitamin B12 transport system, amino acid transport system, and manganese/iron transport system. Modules enriched in controls included aminoacyl-tRNA biosynthesis, coenzyme A biosynthesis, nucleotide synthesis, and dicarboxylate-hydroxybutyrate cycle.

Among postmenopausal women, 43 KEGG modules were enriched in patients, including lipopolysaccharide biosynthesis, iron complex transport system, vitamin B12 transport system, PTS system, secretion system, amino acid transport system, and beta-oxidation (*q* value < 0.05, Wilcoxon rank sum test, Fig. 7b; Additional file 1: Table S10).

The genes annotated to butanoate metabolism pathways differentially enriched in gut microbiome of postmenopausal patients and controls. Fourteen butyrate-synthesis genes were found: 10 genes were enriched in controls and 4 genes were enriched in postmenopausal patients (*q* value < 0.05, Wilcoxon rank sum test; Additional file 1: Table S11).

## Discussion

Here, we performed a comprehensive metagenomic comparison of gut microbiota in breast cancer patients and healthy controls. Microbiota were analyzed in terms of taxonomic profile, genetic functional capacity, and associations with clinical indices of breast cancer. Our results identify various compositional and functional features of the gut microbiota metagenome that differ between postmenopausal patients and healthy controls, suggesting that they may be associated with postmenopausal breast cancer.

Significant taxonomic differences in gut microbiota were not detected between premenopausal breast cancer patients and controls. In contrast, several bacterial species were found to be enriched in postmenopausal patients relative to controls: *Escherichia coli*, *Citrobacter koseri*, *Acinetobacter radioresistens*, *Enterococcus gallinarum*, *Shewanella putrefaciens*, *Erwinia amylovora*, *Actinomyces* sp. *HPA0247*, *Salmonella enterica*, and *Fusobacterium nucleatum*. These results are consistent with previous research on several of these species, which has suggested associations with breast cancer [24]. In addition, we found a weak positive correlation of *Shewanella putrefaciens* and *Erwinia amylovora* with estradiol (*P* < 0.05), although there is no statistical significance after correction for multiple testing (*q* value > 0.05), which may be related to the small sample, but the association can still be considered exploratory. These results are consistent with the idea that the gut microbiota can influence or be affected by estrogen metabolism and thereby provide an independent biomarker of breast cancer. Recent literature has demonstrated that the gut microbiota is the modulation of systemic estrogens [40–42]. Elevated levels of circulating estrogens are associated with an increased risk of breast cancer [43–47].

We found that *Eubacterium eligens* and *Roseburia inulinivorans* were less abundant in postmenopausal breast cancer patients than in postmenopausal controls. *Roseburia inulinivorans* produces butyrate [48, 49]. In order to explore a potential association between butyrate-producing bacteria and breast cancer, we had added a differentially enriched gene analysis to show the potential association, all differentially enriched genes were annotated to the butanoate metabolism pathways, and finally, 14 butyrate-synthesis genes were found: 10 genes were enriched in controls and 4 genes were enriched in postmenopausal patients. Notably, these butyrate-synthesis genes were reduced in postmenopausal patients, which may be related to the decrease in butyrate-producing bacteria.

Butyrate acts as an anti-inflammatory agent, mainly by inhibiting the activation of nuclear factor κB (NF-κB) in intestinal epithelial cells [50]. Butyrate can also act on immune cells via specific G-protein-coupled receptors expressed on immune cells [51]. Reductions in colonic butyrate can promote inflammation. These findings provide further evidence for the idea that alterations in the gut microbial community are associated with breast cancer. For example, a decrease in *Roseburia inulinivorans* may render postmenopausal women more prone to inflammation and therefore at higher risk of breast cancer if the decrease in *Roseburia* had occurred at the time breast cancer was initiated.

Dysbiosis was detected in the gut microbiomes of postmenopausal breast cancer patients, but it was not detected in premenopausal patients. Therefore, the dysbiosis observed in the gut microbiomes of breast cancer patients may depend on age and menopause status.

Breast cancer-associated alterations in the gut microbial community likely translate into alterations in gut microbial functions. Among premenopausal women, breast cancer was associated in our study with enrichment in genes involved in the PTS system, secretion system, vitamin B12 transport system, and manganese/iron transport system. Among postmenopausal women, breast cancer was associated with enrichment in genes involved in lipopolysaccharide (LPS) biosynthesis, iron complex transport system, vitamin B12 transport system, PTS system, and secretion system. Iron enrichment affects the gut microbiome, increases pathogen abundance, and induces intestinal inflammation [52]. The PTS and secretion systems are associated with diabetes, liver cirrhosis, and rheumatoid arthritis [38, 53], while vitamin B12 status correlates positively with breast cancer risk in women [54]. Lipopolysaccharide is a potent trigger of macrophage-mediated systemic inflammation [55], which has been suggested to play an important role in promoting the transformation of inflammation into tumorigenesis [56–58]. Enrichment of the iron transport system and lipopolysaccharide biosynthesis in gut microbiota may cause systemic low-grade inflammation, thereby increasing the risk of breast cancer if the dysbiosis observed in the patient cohort was present in the same cohort prior to contracting breast cancer.

## Conclusion

In conclusion, we have found alterations of gut microbial community and functions in postmenopausal breast cancer patients. The gut microbiota may regulate or respond to host immunity and metabolic balance. In this way, our study suggests an association between gut microbiota and development of postmenopausal breast cancer. However, our data do not allow us to determine whether the altered gut metagenome is the consequence of the disease process or is somehow involved in its pathogenesis.

## Additional files

Additional file 1: **Table S1.** Generated data of the four groups. **Table S2.** Distribution of the samples of the four groups in the two enterotypes. **Table S3.** Relative abundance of the different species between premenopausal breast cancer patients and premenopausal healthy controls. **Table S4.** Relative abundance of the different species between postmenopausal breast cancer patients and postmenopausal healthy controls. **Table S5.** PERMANOVA analysis was performed to assess effects of different phenotypes on gene profile. **Table S6.** The optimal species markers in the classification of postmenopausal breast cancer patients and postmenopausal healthy controls. **Table S7.** The abundance of Pathogen-Host Interactions (PHI) gene coding for diseases in postmenopausal breast cancer patients and postmenopausal healthy controls. **Table S8.** The virulence factor in samples of postmenopausal breast cancer patients and postmenopausal healthy controls. **Table S9.** Relative abundance of the different KEGG modules between premenopausal breast cancer patients and premenopausal healthy controls. **Table S10.** Relative abundance of the different KEGG modules between postmenopausal breast cancer patients and postmenopausal healthy controls. **Table S11.** Differentially enriched genes which annotated to butanoate metabolism pathways between postmenopausal breast cancer patients and postmenopausal healthy controls. **Table S12.** Relative abundance of the species of all the samples. **Table S13.** The species counts of all the samples. (XLS 2530 kb)

Additional file 2: **Figure S1.** Rarefaction for gut microbial gene in premenopausal breast cancer patients (*n* = 18), premenopausal healthy controls (*n* = 25), postmenopausal breast cancer patients (*n* = 44), and postmenopausal healthy controls (*n* = 46). Group 1 indicates premenopausal healthy controls, group 2 indicates premenopausal breast cancer patients, group 3 indicates postmenopausal healthy controls, and group 4 indicates postmenopausal breast cancer patients. **Figure S2.** The enterotypes of gut microbiota in breast cancer patients and healthy controls. (a) The optimal number of enterotypes was two of the four groups as indicated by Calinski-Harabasz (CH) index. The maximum CH index at two clusters (enterotypes) indicated the optimal enterotype number. (b) The gut microbiota of the four cohorts are clustered into two enterotypes at the genus level, dominated by either Bacteroides (enterotype 1) or Prevotella (enterotype 2). (c) Relative abundances of the top genera in the two enterotypes. (d) Distribution of the samples of the four groups in the two enterotypes. **Figure S3.** Relative abundance of the gut microbiota in the four groups at the phylum level. **Figure S4.** Relative abundance of the gut microbiota in the four groups at the genus level. **Figure S5**. Abundance distribution of the gut microbiota differed significantly between postmenopausal breast cancer patients and postmenopausal healthy controls at the genus level. **Figure S6.** Distribution of five trials of tenfold cross-validation error in random forest classification of postmenopausal breast cancer patients. The model was trained using the relative species abundances in patients and controls. The black line marks the average of the five trials (gray lines). The red line indicates the number of optimal species markers. **Figure S7.** Scatter plots for correlations between gut microbiota species and clinical indices. (DOCX 810 kb)
