# Supplementary material for: Breast cancer in postmenopausal women is associated with an altered gut metagenome
Source: Microbiome. 2018 Aug 6;6:136. doi: 10.1186/s40168-018-0515-3 (PMC6080540; doi:10.1186/s40168-018-0515-3)
Supplement: Supplementary file 2 — Figure S1. Rarefaction for gut microbial gene in premenopausal breast cancer patients (n = 18), premenopausal healthy controls (n = 25), postmenopausal breast cancer patients (n = 44), and postmenopausal healthy controls (n = 46). Group 1 indicates premenopausal healthy controls, group 2 indicates premenopausal breast cancer patients, group 3 indicates postmenopausal healthy controls, and group 4 indicates postmenopausal breast cancer patients. Figure S2. The enterotypes of gut microbiota in breast cancer patients and healthy controls. (a) The optimal number of enterotypes was two of the four groups as indicated by Calinski-Harabasz (CH) index. The maximum CH index at two clusters (enterotypes) indicated the optimal enterotype number. (b) The gut microbiota of the four cohorts are clustered into two enterotypes at the genus level, dominated by either Bacteroides (enterotype 1) or Prevotella (enterotype 2). (c) Relative abundances of the top genera in the two enterotypes. (d) Distribution of the samples of the four groups in the two enterotypes. Figure S3. Relative abundance of the gut microbiota in the four groups at the phylum level. Figure S4. Relative abundance of the gut microbiota in the four groups at the genus level. Figure S5. Abundance distribution of the gut microbiota differed significantly between postmenopausal breast cancer patients and postmenopausal healthy controls at the genus level. Figure S6. Distribution of five trials of tenfold cross-validation error in random forest classification of postmenopausal breast cancer patients. The model was trained using the relative species abundances in patients and controls. The black line marks the average of the five trials (gray lines). The red line indicates the number of optimal species markers. Figure S7. Scatter plots for correlations between gut microbiota species and clinical indices. (DOCX 810 kb) [file 40168_2018_515_MOESM2_ESM.docx]

**Additional file 2**

**Supplementary Figures**

**Breast cancer in postmenopausal women is associated with an altered gut metagenome**


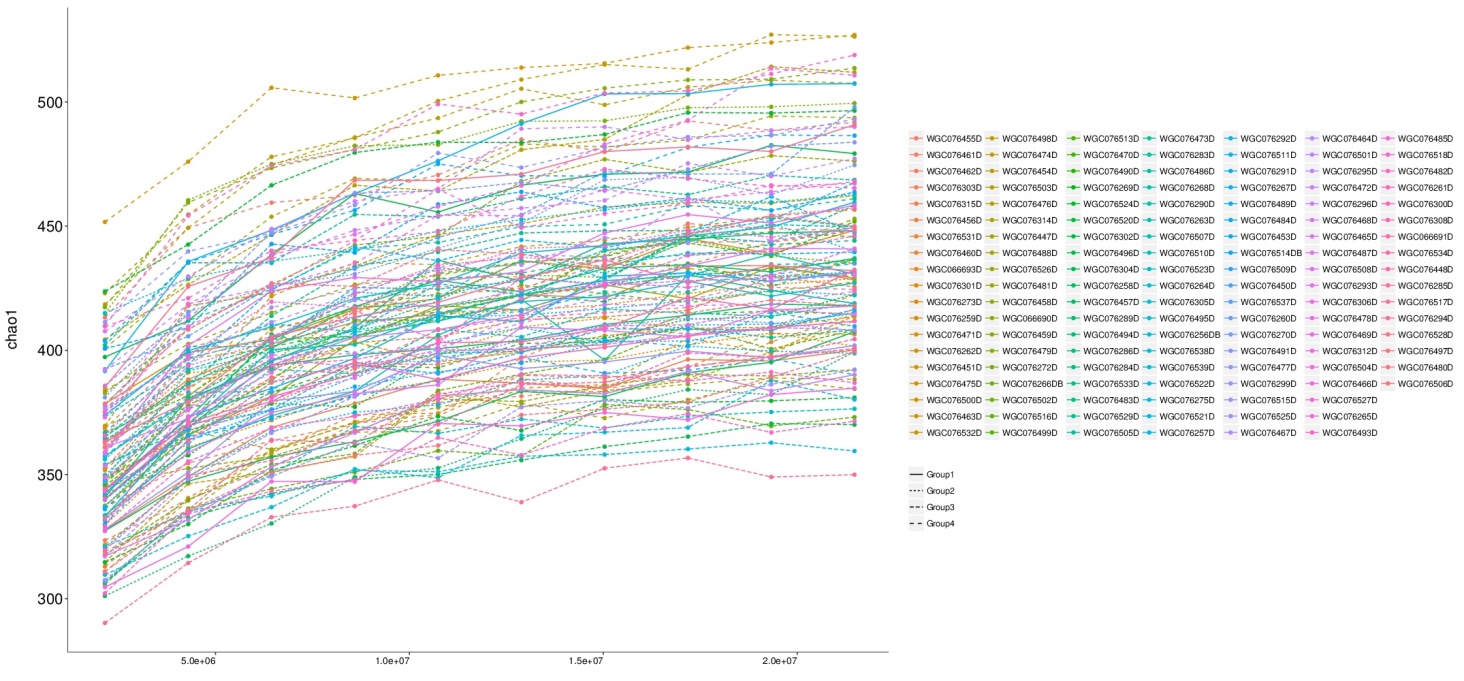


**Figure S1,** Rarefaction for gut microbial gene in premenopausal breast cancer patients (n=18), premenopausal healthy controls (n=25), postmenopausal breast cancer patients (n=44) and postmenopausal healthy controls (n=46). Group 1 indicates premenopausal healthy controls, group 2 indicates premenopausal breast cancer patients, group 3 indicates postmenopausal healthy controls, group 4 indicates postmenopausal breast cancer patients.

b

a


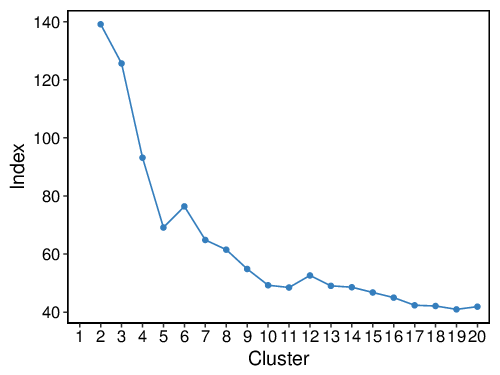

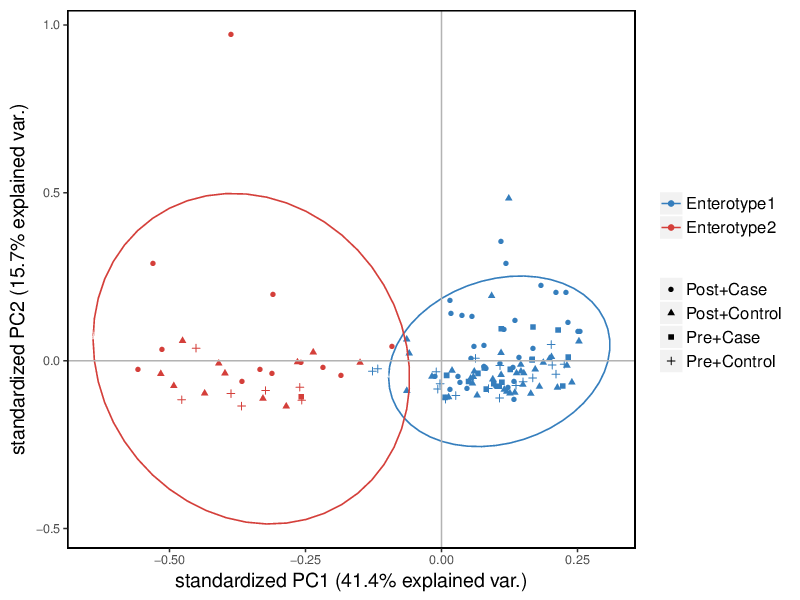


d

c


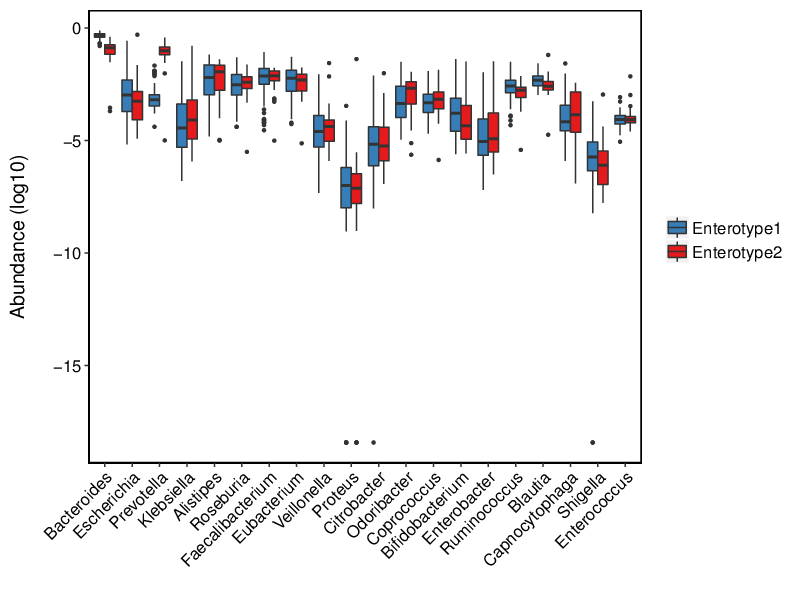

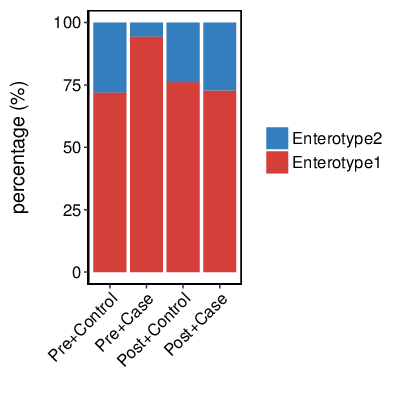


**Figure S2,** The enterotypes of gut microbiota in breast cancer patients and healthy controls. (a) The optimal number of enterotypes was two of the four groups as indicated by Calinski-Harabasz (CH) index. The maximum CH index at two clusters (enterotypes) indicated the optimal enterotype number. (b) The gut microbiota of the four cohorts are clustered into two enterotypes at the genus level, dominated by either Bacteroides (enterotype 1) or Prevotella (enterotype 2). (c) Relative abundances of the top genera in the two enterotypes. (d) Distribution of the samples of the four groups in the two enterotypes.


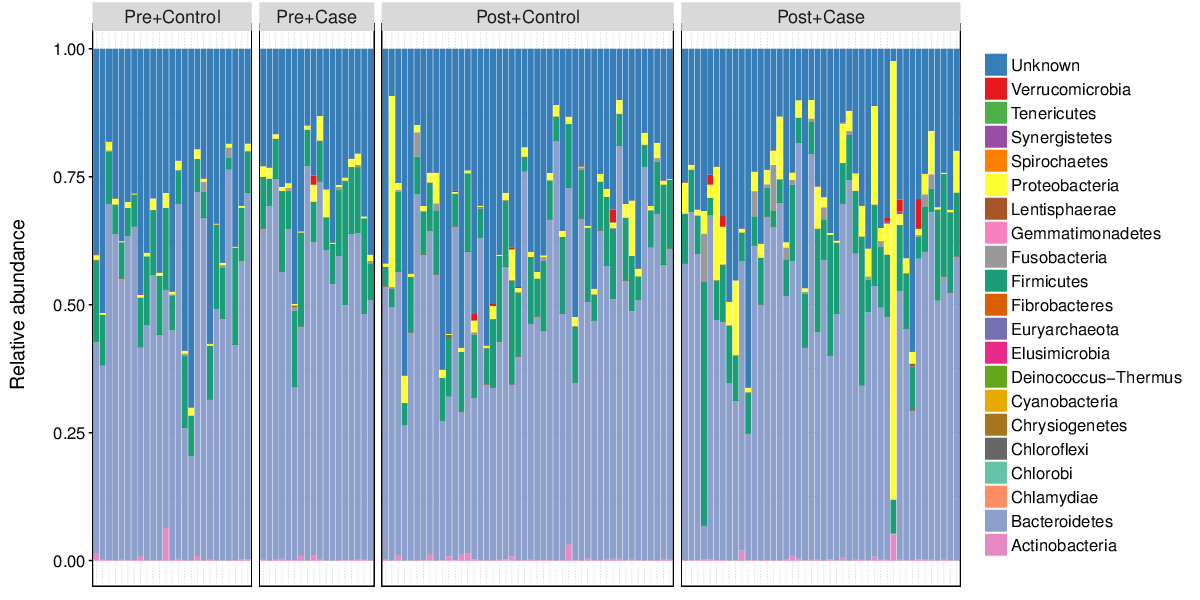


**Figure S3,** Relative abundance of the gut microbiota in the four groups at the phylum level.


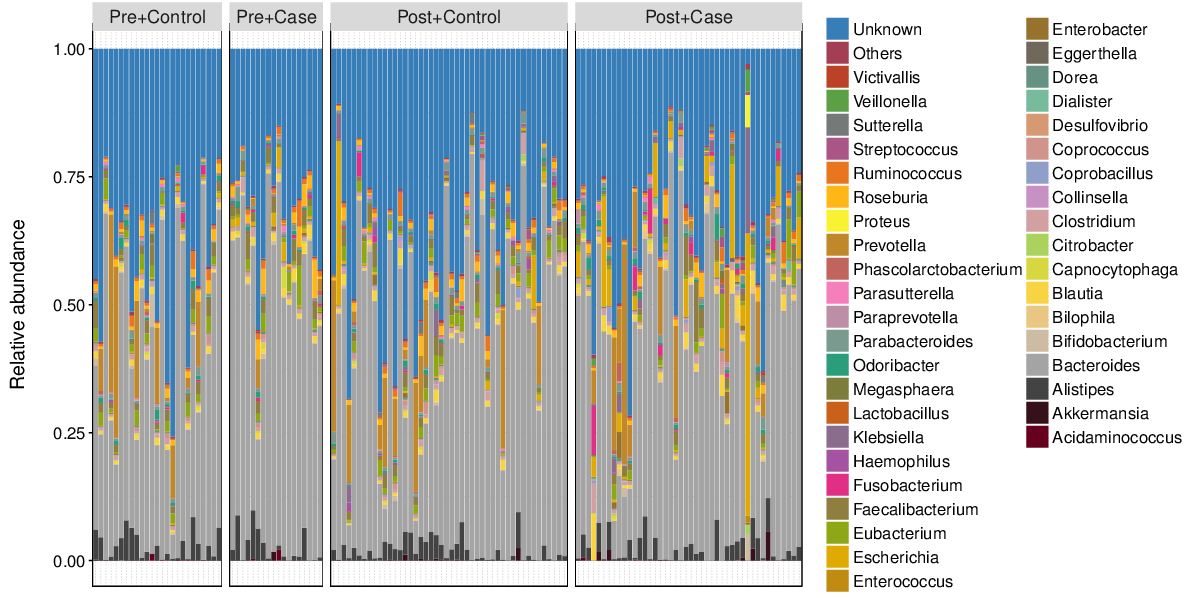


**Figure S4**, Relative abundance of the gut microbiota in the four groups at the genus level.


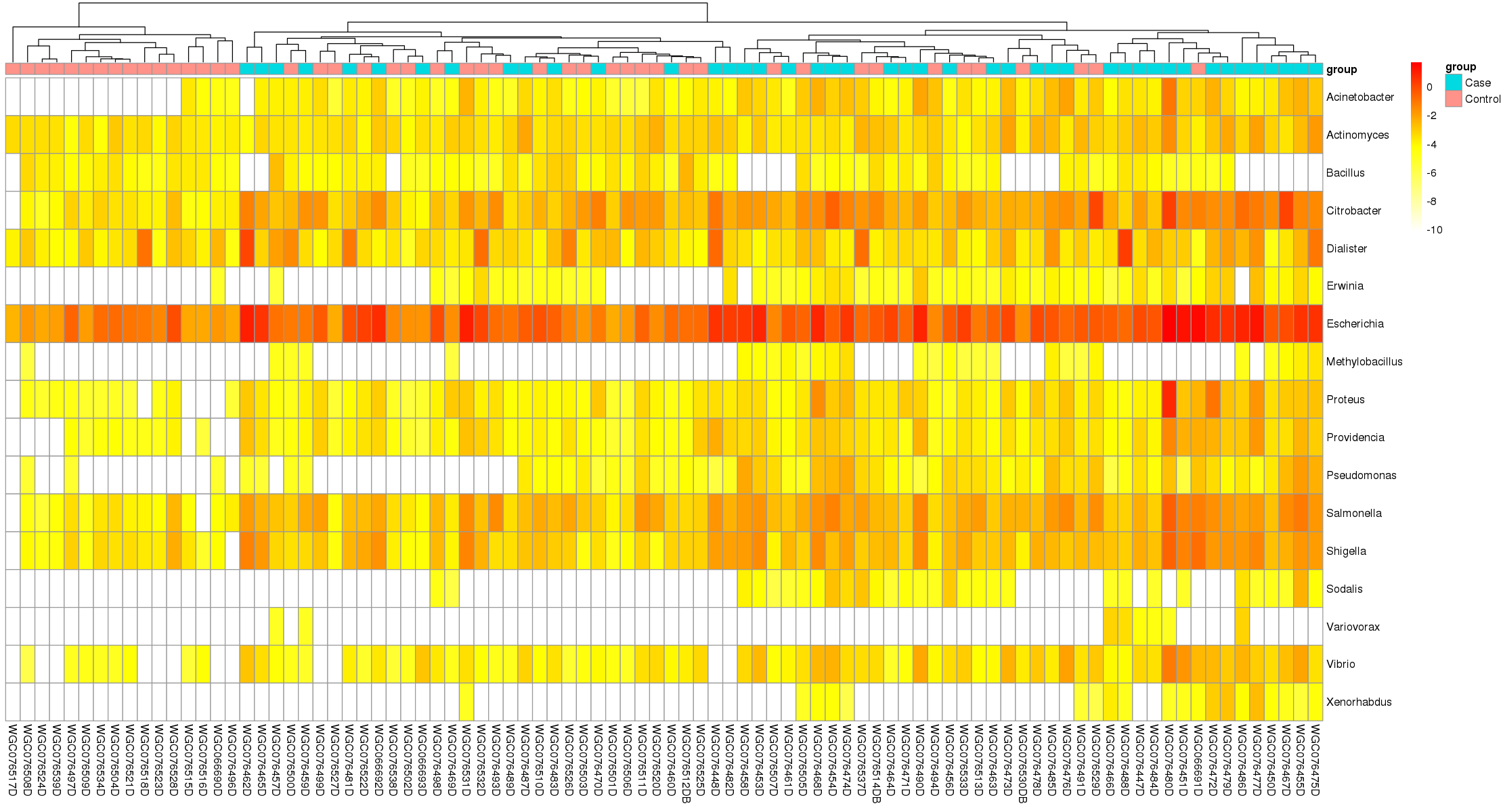


**Figure S5,** Abundance distribution of the gut microbiota differed significantly between postmenopausal breast cancer patients and postmenopausal healthy controls at the genus level.


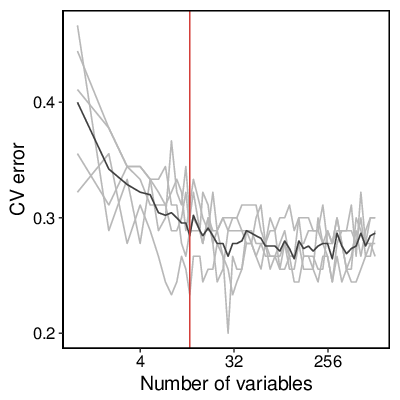


**Figure S6,** Distribution of five trials of 10-fold cross-validation error in random forest classification of postmenopausal breast cancer patients. The model was trained using the relative species abundances in patients and controls. The black line marks the average of the five trials (grey lines). The red line indicates the number of optimal species markers.

Acinetobacter radioresistens Acinetobacter radioresistens


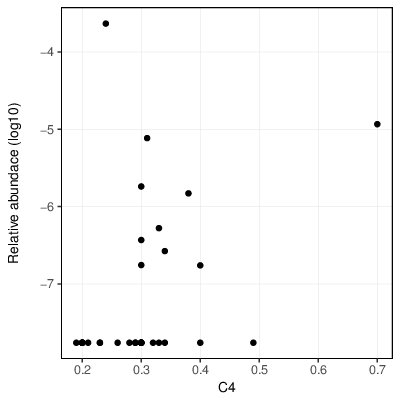

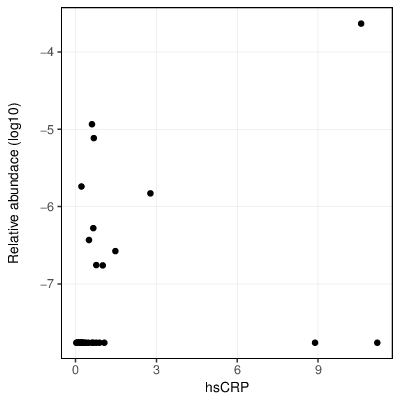


Spearman Rho = 0.413, *P* = 0.015, q-value > 0.05 Spearman Rho = 0.442, *P* = 0.009, q-value > 0.05

Actinomyces sp_HPA0247 Anaerococcus vaginalis


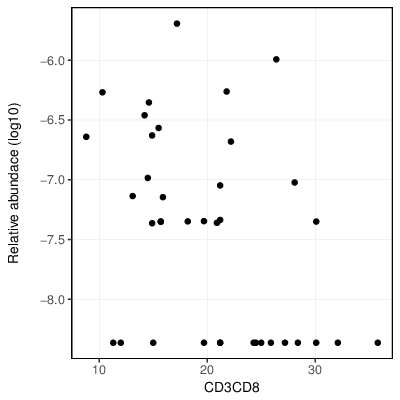

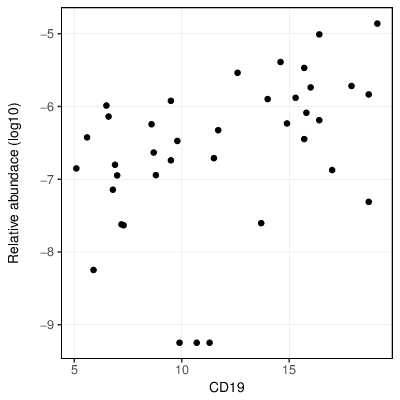


Spearman Rho = ￚ0.384, *P* = 0.017, q-value > 0.05 Spearman Rho = 0.48, *P* = 0.002, q-value > 0.05

Enterococcus gallinarum Porphyromonas uenonis


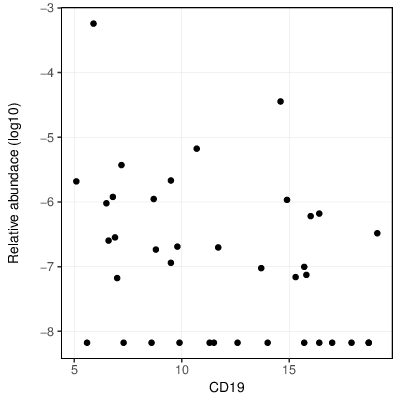

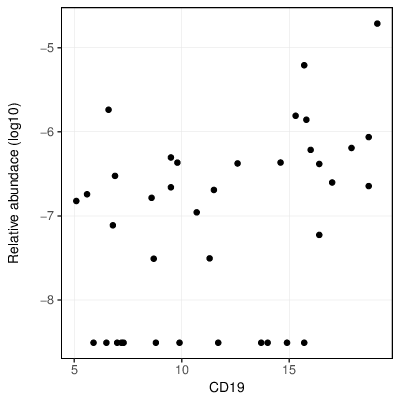


Spearman Rho = ￚ0.351, *P* = 0.031, q-value > 0.05 Spearman Rho = 0.42, *P* = 0.009, q-value > 0.05

Erwinia amylovora Shewanella putrefaciens


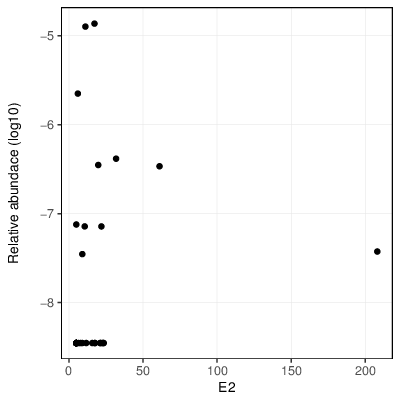

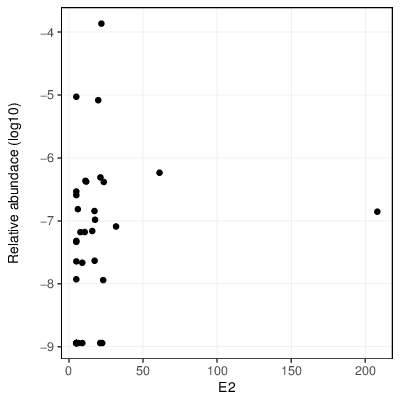


Spearman Rho = 0.351, *P* = 0.039, q-value > 0.05 Spearman Rho = 0.379, *P* = 0.025, q-value > 0.05

Eubacterium eligens Eubacterium eligens


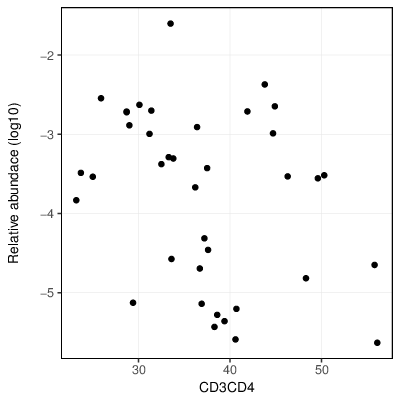

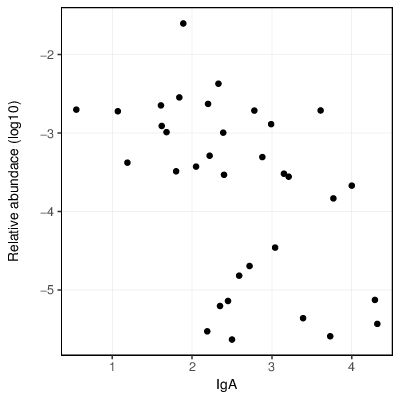


Spearman Rho = ￚ0.349, *P* = 0.032, q-value > 0.05 Spearman Rho = ￚ0.532, *P* = 0.001, q-value > 0.05

Vibrio cholerae Yersinia enterocolitica


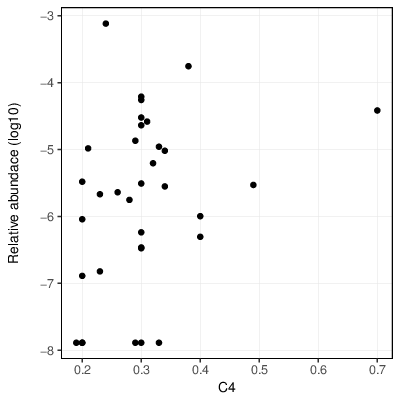

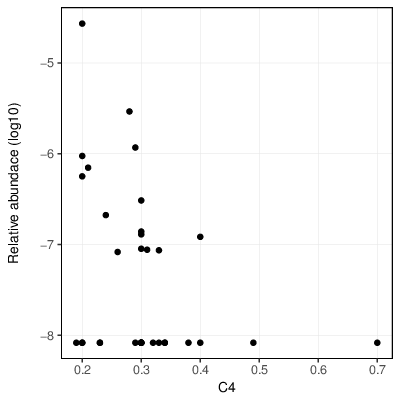


Spearman Rho = 0.349, *P* = 0.043, q-value > 0.05 Spearman Rho = ￚ0.345, *P* = 0.046, q-value > 0.05

Enterococcus gallinarum


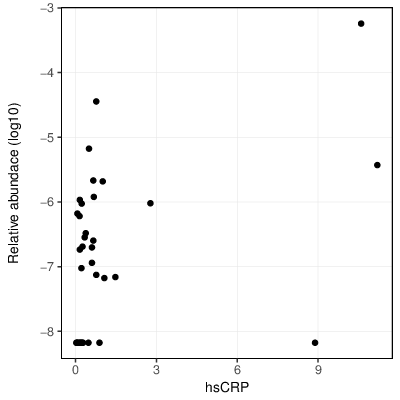


Spearman Rho = 0.386, *P* = 0.024, q-value > 0.05

**Figure S7,** Scatter plots for correlations between gut microbiota species and clinical indices.
